# SARS-CoV-2 vaccination modelling for safe surgery to save lives: data from an international prospective cohort study

**DOI:** 10.1093/bjs/znab101

**Published:** 2021-03-24

**Authors:** COVIDSurg Collaborative, GlobalSurg Collaborative

## Abstract

**Background:**

Preoperative SARS-CoV-2 vaccination could support safer elective surgery. Vaccine numbers are limited so this study aimed to inform their prioritization by modelling.

**Methods:**

The primary outcome was the number needed to vaccinate (NNV) to prevent one COVID-19-related death in 1 year. NNVs were based on postoperative SARS-CoV-2 rates and mortality in an international cohort study (surgical patients), and community SARS-CoV-2 incidence and case fatality data (general population). NNV estimates were stratified by age (18–49, 50–69, 70 or more years) and type of surgery. Best- and worst-case scenarios were used to describe uncertainty.

**Results:**

NNVs were more favourable in surgical patients than the general population. The most favourable NNVs were in patients aged 70 years or more needing cancer surgery (351; best case 196, worst case 816) or non-cancer surgery (733; best case 407, worst case 1664). Both exceeded the NNV in the general population (1840; best case 1196, worst case 3066). NNVs for surgical patients remained favourable at a range of SARS-CoV-2 incidence rates in sensitivity analysis modelling. Globally, prioritizing preoperative vaccination of patients needing elective surgery ahead of the general population could prevent an additional 58 687 (best case 115 007, worst case 20 177) COVID-19-related deaths in 1 year.

**Conclusion:**

As global roll out of SARS-CoV-2 vaccination proceeds, patients needing elective surgery should be prioritized ahead of the general population.

## Teaser

The aim of this study was to inform vaccination prioritization by modelling the impact of vaccination on elective inpatient surgery. The study found that patients aged at least 70 years needing elective surgery should be prioritized alongside other high-risk groups during early vaccination programmes. Once vaccines are rolled out to younger populations, prioritizing surgical patients is advantageous.

## Introduction

The SARS-CoV-2 pandemic has disrupted elective surgery globally, with millions of elective operations either postponed or cancelled^1–3^. Restriction of elective surgery activity was necessary during periods of high COVID-19 hospital admissions in order to divert hospital resources to ICUs^4–6^. However, in many countries, owing to safety concerns, reductions in COVID-19 admissions have been associated with only limited recovery of surgical services^1^. Even in areas with low community SARS-CoV-2 infection rates, surgical patients are at risk of nosocomial SARS-CoV-2 infection^7^, which is associated with high rates of postoperative pulmonary complications and death^8^^,^^9^. Although mitigation measures, such as preoperative reverse transcription (RT)–PCR swab screening and COVID-free surgical pathways, can reduce the risk of COVID-19 complications^7^^,^^10^, they are unlikely to be implemented universally, particularly in low- and middle-income countries (LMICs).

Several SARS-CoV-2 vaccines have been authorized internationally following phase III trials that demonstrated 100 per cent effectiveness in preventing COVID-19-related deaths^11–14^. Preoperative vaccination could support safe reinitiation of elective surgery by significantly reducing the risk of COVID-19 complications in patients undergoing elective procedures. However, it is projected that the population in only 37 countries will have widespread access to vaccination in 2021, with most LMICs only achieving widespread coverage from late 2022 onwards^15^^,^^16^. Consequently, throughout 2021, most governments will prioritize access to vaccination to patients at greatest risk of COVID-19 mortality.

The benefits of SARS-CoV-2 vaccination in surgical patients are currently unknown, and therefore governments are not prioritizing them for vaccination. The aim of this study was to inform vaccination prioritization by modelling the impact of vaccination on mortality in patients undergoing any type of inpatient elective surgery.

## Methods

This study explored the impact of SARS-CoV-2 vaccination in adult patients (age at least 18 years) undergoing any type of elective inpatient surgery. The primary outcome was number needed to vaccinate (NNV) to prevent one COVID-19-related death over 1 year after SARS-CoV-2 vaccination, assuming that surgical patients would receive the vaccination before operation. When comparing NNV values for two different groups, the lower NNV value is more favourable, as this indicates that fewer people in that group need to be vaccinated in order to prevent one death. The secondary outcomes were NNV to prevent one COVID-19-related death over 30 days after SARS-CoV-2 vaccination, and additional COVID-19-related deaths prevented by prioritizing preoperative vaccination for surgical patients versus age-matched controls.

As SARS-CoV-2 mortality is strongly associated with age^17–19^, NNV estimates were preplanned to be stratified by age group (18–49 years, 50–69 years, 70 years or more). In addition, as prioritization and planning of cancer and non-cancer surgery differs, NNV estimates were also preplanned to be stratified by indication for surgery (cancer *versus* non-cancer). To contextualize the NNV estimates for surgical patients, age-stratified NNVs were also estimated for the general population. The study was registered at Clinical.Trials.gov (NCT0450998690).

### Modelling strategy

NNVs in surgical patients were based on postoperative SARS-CoV-2 rates and mortality from the multinational multicentre GlobalSurg–CovidSurg Week study. NNVs in the general population were based on global community SARS-CoV-2 incidence and UK SARS-CoV-2 case fatality data. It was assumed that, outside of the initial 30-day postoperative window, during the following 335 days surgical patients are at the same risk of SARS-CoV-2 infection and SARS-CoV-2 mortality as the general population within the same age group.

The following equation was used to calculate NNV to prevent one COVID-19-related death over 1 year after SARS-CoV-2 vaccination in the surgical groups:

NNV=1/(S+(D×335×C))

where S is postoperative mortality (within 30 days after surgery) attributable to SARS-CoV-2 infection, D is the daily community SARS-CoV-2 incidence, and C is the case fatality rate for SARS-CoV-2 in the general population.

To calculate the NNV to prevent one death over 1 year after vaccination in the general population, the equation was:

NNV=1/(D×365×C).

### GlobalSurg–CovidSurg Week study

Real-world estimates for postoperative SARS-CoV-2 rates and postoperative mortality attributable to SARS-CoV-2 were based on the GlobalSurg-CovidSurg Week study. This was an international prospective multicentre cohort study (NCT04509986) that included patients undergoing any type of surgical procedure performed routinely in an operating theatre by a surgeon. Participating centres collected data on all consecutive patients in one or more preselected surgical specialties during 1 or more weeks within an overall 28-day study window (5 October 2020 to 1 November 2020). Patients who had either elective surgery (planned admission to hospital) or emergency surgery (unplanned admission), as either an inpatient (surgery with a planned overnight stay) or day case, were included. This analysis was restricted to patients having elective inpatient surgery. Mortality status was determined by 30-day postoperative follow-up. Patients with data missing on age, sex, ASA physical status grade, indication for surgery, grade of surgery, SARS-CoV-2 infection status, or mortality were excluded from this analysis, to allow consistent denominators throughout calculations.

The study collected only routine, anonymized data with no change to clinical care pathways. A secure online REDCap database was used for data collection. The study was registered at each participating hospital in line with applicable regulations. In the UK, the study was registered as either a clinical audit or service evaluation (registration reference at lead site, University Hospitals Birmingham NHS Foundation Trust: CARMS-16328).

### Postoperative mortality attributable to SARS-CoV-2

The proportion of patients who died within 30 days of surgery with death attributable to a postoperative SARS-CoV-2 infection was calculated as I × R, where I is the postoperative SARS-CoV-2 rate in the first 30 days after surgery, and R is the postoperative SARS-CoV-2 attributable mortality (an adjusted estimate for the difference in mortality between patients who did and did not have postoperative SARS-CoV-2 infection).

### Postoperative SARS-CoV-2 rates

In the GlobalSurg–CovidSurg Week study, postoperative SARS-CoV-2 was defined as any diagnosis of SARS-CoV-2 made after surgery, up to and including postoperative day 30. This included both patients with and without symptoms. SARS-CoV-2 could be diagnosed based on a positive RT–PCR swab, positive rapid antigen test, positive chest CT, and/or contemporaneous clinical diagnosis (in the absence of negative RT–PCR swab results). Patients who had a diagnosis of SARS-CoV-2 at any time before surgery were excluded from the denominator for calculating postoperative SARS-CoV-2 rates.

### Postoperative SARS-CoV-2 attributable mortality

Multiple studies have established that patients who become infected with SARS-CoV-2 after operation are at increased risk of postoperative death^8^^,^^9^. However, there are likely to be confounding factors, with higher postoperative mortality rates in SARS-CoV-2-infected patients partly attributable to baseline differences such as older age or co-morbidity. Therefore, an unadjusted risk difference for mortality between patients with and without postoperative SARS-CoV-2 infection is likely to overestimate mortality attributable to SARS-CoV-2.

To reduce confounding, data for elective inpatient surgery from the GlobalSurg–CovidSurg Week study were used to estimate adjusted differences in mortality between patients who did and did not have postoperative SARS-CoV-2. This was based on average marginal effects taken from multilevel logistic regression models. The outcome in the models was 30-day mortality. As well as postoperative SARS-CoV-2 infection, the models were adjusted for factors that have previously been identified as independent predictors of death in patients with perioperative SARS-CoV-2 infection^8^: age, sex, ASA physical status grade (grades I–II *versus* III–V), indication (surgery for benign *versus* malignant disease), and grade of surgery (minor *versus* major, based on the BUPA schedule of procedures^20^). In addition, country was included as a random effect, with hospital nested within country. Models were stratified by age subgroup (20–49 years, 50–69 years, 70 years or more). Analyses were completed in Stata^®^ version 15.1 (StataCorp, College Station, Texas, USA).

### Daily community SARS-CoV-2 incidence

Daily community SARS-CoV-2 incidence was based on SARS-CoV-2 cases reported globally in 2020^21^. Countries were split into tertiles by community SARS-CoV-2 incidence rank. Median daily community SARS-CoV-2 incidence was calculated for each tertile to produce values for low, medium, and high community SARS-CoV-2 incidence. The main analysis was based on medium SARS-CoV-2 incidence.

### Case fatality rates in the general population

Age-stratified SARS-CoV-2 case fatality rates in the general population were calculated using SARS-CoV-2 seroprevalence and mortality data from England. The full methodology is described in *Appendix S1*.

### Vaccine effectiveness

No deaths from COVID-19 have been reported beyond 1 week after SARS-CoV-2 vaccination in the phase III trials published to date^11–14^. The main analysis was therefore based on SARS-CoV-2 vaccination being 95 per cent effective in preventing death from COVID-19.

### COVID-19-related deaths prevented

The number of additional COVID-19-related deaths that could be prevented in 1 year by vaccinating surgical patients before operation rather than the general population was calculated by subtracting the number of COVID-19-related deaths that would occur over 30 days in the unvaccinated general population from the number of COVID-19-related deaths that would occur in first 30 postoperative days in unvaccinated surgical patients. Estimates were age-stratified (*Appendix S1*).

### Sensitivity analyses for community SARS-CoV-2 incidence

NNV values vary depending on local SARS-CoV-2 incidence. Therefore, a significant area of uncertainty concerns SARS-CoV-2 incidence in the general population, as this varies across both geographical regions and time. Future variation may reflect changes in government policies, or the emergence of new viral strains with either increased or decreased transmissibility. One-way sensitivity analyses were therefore undertaken, applying the low and high community SARS-CoV-2 incidence estimates.

### Best- and worst-case scenarios

Best- and worst-case scenarios were produced to further explore uncertainty. The best-case scenario represents the lowest likely value for NNV and the highest likely value for additional COVID-19-related deaths prevented, whereas the worst-case scenario represents the highest likely value for NNV and the lowest likely value for additional COVID-19-related deaths prevented. The parameters for the best- and worst-case scenarios are described in *Appendix S1* and summarized in *Table 1*.

**Table 1:** Parameters used for main analysis, and best- and worst-case scenarios

	**Worst-case s**ce**nario**	Main analysis	**Best-case s**ce**nario**
Postoperative SARS-CoV-2 rates	Based on lower bound of 95% confidence interval for 30-day postoperative SARS-CoV-2 rates	Based on point estimate for 30-day postoperative SARS-CoV-2 rates	Based on upper bound of 95% confidence interval for 30-day postoperative SARS-CoV-2 rates
Postoperative SARS-CoV-2 attributable mortality^*^	Based on lower bound of 95% confidence interval for adjusted difference in 30-day mortality	Based on point estimate for adjusted difference in 30-day mortality	Based on upper bound of 95% confidence interval for adjusted difference in 30-day mortality
Community SARS-CoV-2 infection rates	All scenarios modelled based on medium SARS-CoV-2 incidence. Separate one-way sensitivity analyses performed for low and high SARS-CoV-2 incidence (other parameters in these sensitivity analyses are based on main analysis)		
Community SARS-CoV-2 case fatality rate	Based on upper bound of 95% credible intervals published by ONS for new SARS-CoV-2 cases per day	Based on point estimate published by ONS for new SARS-CoV-2 cases per day	Based on lower bound of 95% credible intervals published by ONS for new SARS-CoV-2 cases per day
SARS-CoV-2 vaccine effectiveness	Based on vaccination having 100% effectiveness in preventing COVID-19-related deaths	Based on vaccination having 95% effectiveness in preventing COVID-19-related deaths	Based on vaccination having 80% effectiveness in preventing COVID-19-related deaths

* Based on adjusted differences in 30-day mortality between patients with and without postoperative SARS-CoV-2 infection. ONS, Office for National Statistics.

## Results

### GlobalSurg–CovidSurg week study

Overall, the GlobalSurg–CovidSurg Week study captured data for 141 582 patients from across 1667 hospitals in 116 countries. Of the 89 225 adults who underwent elective surgery, 31 434 had a day-case procedure and 1202 had a preoperative SARS-CoV-2 infection. Therefore, 56 589 patients were included in the main analyses (*Fig. S1*). A demographic breakdown is provided in *Table 2*.

**Table 2:** Age-stratified baseline demographics and outcomes for patients who had inpatient elective surgery in the GlobalSurg–CovidSurg Week study

	**18–49 years** (*n* = 21 836)	**50–69 years** (*n* = 21 577)	**≥70 years** (*n* = 13 176)	***P*** *
**Age (years)**				< 0.001
18–29	5512 (25.2)	–	–	
30–39	7898 (36.2)	–	–	
40–49	8426 (38.6)	–	–	
50–59	–	10 204 (47.3)	–	
60–69	–	11 373 (52.7)	–	
70–79	–	–	9491 (72.0)	
≥ 80	–	–	3685 (28.0)	
**Sex**				< 0.001
F	7827 (35.8)	10 959 (50.8)	6148 (46.7)	
M	14009 (64.2)	10 618 (49.2)	7028 (53.3)	
**ASA physical status grade**				< 0.001
I–II	19 373 (88.7)	14 801 (68.6)	6370 (48.3)	
III–V	2463 (11.3)	6776 (31.4)	6806 (51.7)	
**Indication**				< 0.001
Non-cancer surgery	18 241 (83.5)	13 906 (64.4)	7932 (60.2)	
Cancer surgery	3595 (16.5)	7671 (35.6)	5244 (39.8)	
**Grade of surgery**				
Minor	5564 (25.5)	5063 (23.5)	3132 (23.8)	< 0.001
Major	16 272 (74.5)	16 514 (76.5)	10 044 (76.2)	
**Postoperative SARS-CoV-2**				< 0.001
No	21 676 (99.3)	21 347 (98.9)	13 025 (98.9)	
Yes	160 (0.7)	230 (1.1)	151 (1.1)	
**30-day mortality**				< 0.001
No	21 750 (99.6)	21 362 (99.0)	12 946 (98.3)	
Yes	86 (0.4)	215 (1.0)	230 (1.7)	

Values in parentheses are percentages. Patients with a preoperative diagnosis of SARS-CoV-2 were excluded (*Fig. S1*). *χ^2^ test.

### Postoperative SARS-CoV-2 rates

The overall postoperative SARS-CoV-2 rate was 0.96 per cent (541 of 56 589). Incidence by subgroup is shown in *Table S1*. Overall, 504 of 541 diagnoses (93.2 per cent) were based on a positive RT–PCR (495) or positive rapid antigen test (15). A further 19 patients (3.5 per cent) had a positive chest CT, and the remaining 18 (3.3 per cent) had a clinical diagnosis.

### Postoperative SARS-CoV-2 attributable mortality

In adjusted multilevel models, SARS-CoV-2 infection was significantly associated with 30-day postoperative mortality across all age groups (*Table 3*). Adjusted differences in 30-day mortality between patients with and without postoperative SARS-CoV-2 infection were calculated from these models and are presented in *Table 4*.

**Table 3:** Age-stratified adjusted multilevel models for 30-day mortality in elective inpatient surgery patients

	18–49 years		50–69 years		≥70 years	
	Odds ratio	*P*	Odds ratio	*P*	Odds ratio	*P*
**Age (years)**						
18–29	1.00 (reference)		–		–	
30–39	0.69 (0.37, 1.29)	0.239	–		–	
40–49	0.99 (0.56, 1.73)	0.968	–		–	
50–59	–		1.00 (reference)		–	
60–69	–		1.22 (0.91, 1.64)	0.190	–	
70–79	–		–		1.00 (reference)	
≥ 80	–		–		1.66 (1.25, 2.20)	< 0.001
**Sex**						
F	1.00 (reference)		1.00 (reference)		1.00 (reference)	
M	1.45 (0.93, 2.26)	0.102	1.17 (0.87, 1.57)	0.299	1.51 (1.14, 2.01)	0.004
**ASA physical status grade**						
I–II	1.00 (reference)		1.00 (reference)		1.00 (reference)	
III–V	6.69 (4.19, 10.69)	< 0.001	5.24 (3.79, 7.24)	< 0.001	4.44 (3.09, 6.38)	< 0.001
**Indication**						
Non-cancer surgery	1.00 (reference)		1.00 (reference)		1.00 (reference)	
Cancer surgery	3.92 (2.45, 6.27)	< 0.001	2.07 (1.53, 2.82)	< 0.001	1.85 (1.39, 2.46)	< 0.001
**Grade of surgery**						
Minor	1.00 (reference)		1.00 (reference)		1.00 (reference)	
Major	1.19 (0.67, 2.10)	0.546	1.04 (0.72, 1.52)	0.824	1.52 (1.05, 2.19)	0.027
**Postoperative SARSCoV-2**						
No	1.00 (reference)		1.00 (reference)		1.00 (reference)	
Yes	4.07 (1.18, 14.13)	0.027	11.52 (6.30, 21.09)	< 0.001	10.31 (6.18, 17.20)	< 0.001

Values in parentheses are 95 per cent confidence intervals. Separate multilevel models were created for each age group. This analysis was adjusted for age, sex, ASA grade, surgical indication, grade of surgery, and postoperative SARS-CoV-2 infection, with country and hospital effects included. Unadjusted models are shown in *Tables S2–S4*.

**Table 4:** Adjusted differences in 30-day mortality rate between patients with and without postoperative SARS-CoV-2 infection

	Adjusted difference in 30-day mortality rate (%)
**Age 18–49 years**	
Elective non-cancer surgery	0.77 (0, 2.00)
Elective cancer surgery	2.63 (0, 6.60)
**Age 50–69 years**	
Elective non-cancer surgery	7.26 (3.38, 11.14)
Elective cancer surgery	11.55 (6.08, 17.02)
**Age ≥ 70 years**	
Elective non-cancer surgery	10.46 (5.56, 15.36)
Elective cancer surgery	15.84 (9.30, 22.39)

Values in parentheses are 95 per cent confidence intervals; if the lower bound of the 95 per cent confidence interval included negative values, this was reported as 0 per cent. Adjusted differences were calculated using average marginal effects, based on multilevel models (including country and hospital effects) that were adjusted for age, sex, ASA physical status grade, indication for operation (cancer *versus* non-cancer surgery), and grade of surgery (minor *versus* major).

### NNV to prevent one COVID-19-related death over 1 year

**Fig. 1:**
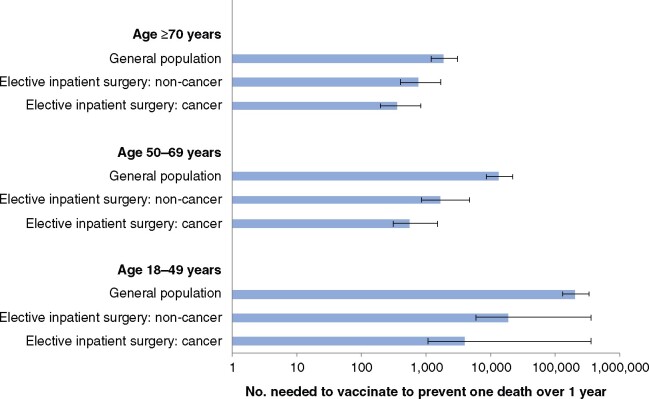
Global number needed to vaccinate to prevent one COVID-19-related death over 1 year Number needed to vaccinate estimates for the general population are based on global SARS-CoV-2 infection rates (26.48 per million people per day); estimates assume that this remains steady for a full year. For surgical patients, estimates are based on preoperative vaccination. Error bars indicate estimates for best- and worst-case scenarios. Data are presented on a logarithmic scale.

In the main analysis, NNVs to prevent one COVID-19-related death over 1 year were lower for surgical patients than the general population in all age groups (*Fig. 1*). NNVs were lowest in people aged at least 70 years: 351 (196 for best-case, 816 for worst-case scenario) among those needing cancer surgery, 733 (407, 1664) for those needing non-cancer surgery, and 1840 (1196, 3066) for the general population. However, NNVs in patients aged 50–69 years needing either cancer surgery (559; 304, 1482) or non-cancer surgery (1621; 854, 4577) were favourable compared with the NNV for the general population aged 70 years or more.

**Fig. 2:**
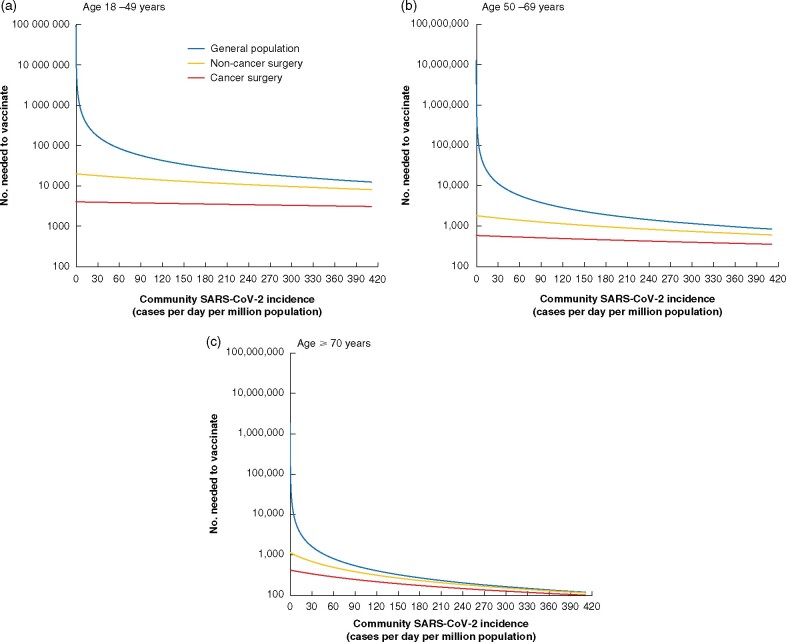
Estimates for number needed to vaccinate to prevent one COVID-19-related death over 1 year, based on country-specific SARS-COV-2 community infection rates, stratified by age **a** 18–49 years, **b** 50–69 years, and **c** 70 years or more. Number needed to vaccinate is plotted on a logarithmic scale.

In sensitivity analyses modelling both low and high community SARS-CoV-2 infection rates, NNVs for surgical patients remained favourable (*Table 5*). Across all countries, there was an advantage to prioritizing surgical patients (*Fig. 2*). Relative to the general population, vaccination of surgical patients had the greatest advantage in countries with low community SARS-CoV-2 infection rates.

**Table 5:** Sensitivity analyses for low, medium, and high community SARS-CoV-2 incidence, showing number needed to vaccinate to prevent one COVID-19-related death over 1 year

	NNV to prevent one death over 1 year		
	18–49 years	50–69 years	≥70 years
**Low SARS-CoV-2 incidence**			
General population	3 378 555 (2 196 461, 5 630 327)	222 028 (144 344, 370 007)	31 692 (20 603, 52 814)
Elective non-cancer surgery	20 049 (6167, 6 134 536)	1819 (936, 5610)	1119 (577, 3135)
Elective cancer surgery	3991 (1094, 6 134 536)	581 (314, 1577)	420 (228, 1060)
**Medium SARS-CoV-2 incidence**			
General population	196 131 (127 509, 326 851)	12 889 (8379, 21 480)	1840 (1196, 3066)
Elective non-cancer surgery	18 421 (5920, 356 121)	1621 (854, 4577)	733 (407, 1644)
Elective cancer surgery	3922 (1086, 356 121)	559 (304, 1482)	351 (196, 816)
**High SARS-CoV-2 incidence**			
General population	43 088 (28 012, 71 806)	2832 (1841, 4719)	404 (263, 674)
Elective non-cancer surgery	14 103 (5142, 78 236)	1150 (641, 2701)	319 (193, 601)
Elective cancer surgery	3682 (1056, 78 236)	490 (272, 1210)	216 (128, 437)

Values in parentheses are results for best- and worst-case scenarios. Estimates assume that the community SARS-CoV-2 incidence remains at a steady rate for a full year. All parameters other than SARS-CoV-2 incidence are based on the main analysis. NNV, number needed to vaccinate.

### NNV to prevent one COVID-19-related death over 30 days

Within each age group, NNVs to prevent one COVID-19-related death over 30 days were lower for surgical patients than for the general population, regardless of community SARS-CoV-2 incidence (*Table 6*). Across age groups, NNVs were lowest in people aged 70 years or more: 425 (231 for best-case, 1080 for worst-case scenario) for those needing cancer surgery, 1157 (592, 3316) for those needing non-cancer surgery, and 22 384 (14 552, 37 302) for the general population.

**Table 6:** Sensitivity analyses for surgical patients needing elective non-cancer or cancer surgery, and low, medium, and high community SARS-CoV-2 incidence in the general population, showing number needed to vaccinate to prevent one COVID-19-related death over 30 days

	NNV to prevent one death over 30 days		
	18–49 years	50–69 years	≥70 years
Surgical patients*			
Elective non-cancer surgery	20 159 (6183, n.a.^†^)	1833 (942, 5689)	1157 (592, 3316)
Elective cancer surgery	3995 (1094, n.a.^†^)	583 (315, 1583)	425 (231, 1080)
**General population**			
Low SARS-CoV-2 incidence	41 105 752 (26 723 608, 68 503 312)	2 701 335 (1 756 188, 4 501 746)	385 581 (250 673, 642 566)
Medium SARS-CoV-2 incidence	2 386 263 (1 551 354, 3 976 683)	156 817 (101 950, 261 335)	22 384 (14 552, 37 302)
High SARS-CoV-2 incidence	524 238 (340 817, 873 637)	34 451 (22 397, 57 413)	4917 (3197, 5689)

Values in parentheses are results for best- and worst-case scenarios. All parameters other than SARS-CoV-2 incidence are based on the main analysis. *A single set of estimates is provided for surgical patients, because SARS-CoV-2 infection rates within 30 days of surgery were modelled from rates observed in the GlobalSurg–CovidSurg Week study, so this was independent of community SARS-CoV-2 rates. ^†^As there is no postoperative mortality attributable to SARS-CoV-2 infection in the worst-case scenario for patients aged 18–49 years (*Table 4*), in the worst-case scenario there would be no benefit from vaccinating these patients. NNV, number needed to vaccinate; n.a., not applicable.

### COVID-19-related deaths prevented

Globally, a policy of preoperative vaccination of patients aged 70 years or more before elective surgery in preference to age-matched general populations was projected to prevent an additional 26 624 (9865 for worse-case, 50 410 for best-case scenario) COVID-19-related deaths in 1 year, assuming that global surgical activity was at 75 per cent of prepandemic volume (*Table S7*). Prioritizing all surgical patients for preoperative vaccination ahead of the general population was projected to prevent an additional 58 687 (20 177, 115 007) COVID-19-related deaths.

## Discussion

This study used real-world data from an international, prospective cohort study to model NNVs to prevent COVID-19-related deaths among patients needing elective inpatient surgery. When compared within age groups, NNVs were consistently lower for surgical patients than for the general population. NNVs were particularly favourable for patients needing cancer surgery, who are likely to be prioritized for elective surgery as services restart in 2021. These findings were consistent across all settings regardless of community SARS-CoV-2 infection rates, as well as in best- and worst-case scenario analyses.

SARS-CoV-2 vaccination could prevent tens of thousands of COVID-19-related postoperative deaths. However, vaccine supplies are likely to remain limited in most countries throughout 2021^16^. Most governments are therefore prioritizing vaccination for groups at highest risk of COVID-19 mortality^22^. This modelling study can inform prioritization of surgical patients within vaccination plans. It supports prioritization of patients aged 70 years or more needing elective surgery alongside other high-risk groups during early vaccination programmes. Once vaccines are rolled out to the wider population, it will be advantageous to prioritize surgical patients, particularly those undergoing cancer surgery.

Implementation of vaccination for surgical patients will require the development of preoperative pathways to deliver vaccination ahead of planned surgery dates. These pathways should be designed alongside wider system developments aimed at reducing nosocomial SARS-COV-2 transmission, such as preoperative SARS-CoV-2 swab testing and COVID-free surgical pathways^7^^,^^10^. Future research should evaluate which of the licensed vaccines is most effective in surgical patients and the optimal timing for preoperative vaccination.

There may be additional benefits to vaccinating surgical patients. Up to 70 per cent of elective procedures were postponed during the first wave of the SARS-CoV-2 pandemic, resulting in an estimated 28 million elective operations being delayed or cancelled^2^^,^^3^. Although elective surgery volumes have started to recover in many countries, ongoing disruption is likely to continue throughout 2021, particularly in the event of countries experiencing further SARS-CoV-2 waves. Prioritization of SARS-CoV-2 vaccination for surgical patients could support safe reinitiation of elective surgery services, especially in regions where vaccinating the total population will take several years. In addition, SARS-CoV-2 vaccination is likely to decrease postoperative pulmonary complications, reducing intensive care use and overall healthcare costs.

This study has limitations. First, NNV estimates should be interpreted with caution. Although NNVs for surgical patients are advantageous compared with those for the general population in sensitivity analyses for both low and high SARS-CoV-2 infection rates, precise NNV values fluctuate depending on prevailing SARS-CoV-2 infection rates; the higher the SARS-CoV-2 incidence, the fewer people need to be vaccinated to prevent one SARS-CoV-2 infection, so the more favourable (lower) the NNV becomes. Second, NNV estimates were based on postoperative SARS-CoV-2 infection rates taken from a global snapshot in October 2020, and it is unknown how these rates vary over time and across regions. Third, SARS-CoV-2 case fatality rates were based on data from England and it is unknown how generalizable these are, although they are broadly consistent with other published data (*Appendix S1*). Finally, vaccine effectiveness was modelled based on the limited data available from trials performed in the general population and it is unknown how applicable this is to surgical patients.

## Supplementary Material

znab101_Supplementary_Data
