# Supplementary material for: SARS-CoV-2 vaccination modelling for safe surgery to save lives: data from an international prospective cohort study
Source: Br J Surg. 2021 Mar 24;108(9):1056–63. doi: 10.1093/bjs/znab101 (PMC7995808; doi:10.1093/bjs/znab101)
Supplement: znab101_Supplementary_Data [file znab101_supplementary_data.zip › Appendix S1 and supplementary tables and figure.docx]

**SARS-CoV-2 vaccination to support safe surgery during the pandemic: a modelling study using data from an international prospective cohort study**

**Appendix S1 Supplementary material**

*Case fatality rate in the general population*

The UK Office for National Statistics (ONS) runs the Coronavirus (COVID-19) Infection Survey, a repeated cross-sectional survey^1^. The survey is based on people from randomly selected private households submitting nose and throat swabs for SARS-CoV-2 analysis (real-time reverse transcriptase polymerase chain reaction, RT-PCR assay). The survey is based on a large sample size, with several hundred thousand individuals submitting swabs for laboratory analysis, including both people with and without symptoms.

Based on the Coronavirus (COVID-19) Infection Survey results, the ONS publishes modelled estimates for new cases per day per 10,000 population^1^. To obtain estimates for total infections, each daily estimate for England was multiplied by the ONS estimate for England's adult (≥18 years) population in 2019 (n=43,004,640)^2^. Daily estimated cases were summed to calculate total cases within the period of interest.

To calculate a case fatality rate, estimates for SARS-CoV-2 incidence were matched to data COVID-19 death statistics. Among patients who die with COVID-19, there is typically a time lag between SARS-CoV-2 diagnosis and death of around 4 weeks^3-5^. Therefore, we matched estimates for SARS-CoV-2 cases to COVID-19 deaths four weeks later. In order to reduce imprecisions in this methodology due to fluctuating SARS-CoV-2 incidence, case data were taken from a period when SARS-CoV-2 incidence was relatively stable in England: 28 May 2020 to 26 August 2020. During this period there was a median of 0.27 per 10,000 population (interquartile range 0.26-0.30) daily new SARS-CoV-2 cases in England.

Based on the ONS incidence point estimates, we calculated that during the 13 weeks from 28 May 2020 to 26 August 2020 there were a total of 115,252 SARS-CoV-2 cases in adults in England. This compares to 64,552 total cases detected during this period in people aged ≥20 years through diagnostic testing in hospitals and the community^6^.

The ONS did not provide age-stratified incidence estimates throughout the full time period 28 May 2020 to 26 August 2020. However, NHS Test & Trace provided a breakdown of the 64,552 cases detected in this period: 38,336 (59.4%) were in people aged 20-49 years, 14,917 (23.1%) were people aged 50-69 years, and 11,299 (17.5%) were in people aged ≥70 years^6^. Applying these ratios to the projected total of 115,252 SARS-CoV-2 cases in England, produced estimates of 68,446 cases in people aged 20-49 years, 26,633 in people aged 50-69 years, and 20,173 cases in people aged ≥70 years. Data were collated for the age group 20-49 years rather than 18-49 years as these are the data cut-offs publicly available.

UK Coronavirus Dashboard^7^ data on deaths within 28 days of SARS-CoV-2 diagnosis were accessed for the period 25 June 2020 to 23 September 2020 (window shifted four weeks forward from that for estimating cases). During this period in England there were 38 deaths within 28 days of SARS-CoV-2 diagnosis in people aged 20-49 years, 227 deaths in people aged 60-69 years, and 1,194 deaths in people aged ≥70 years.

Based on the above figures, the case fatality rate for SARS-CoV-2 in the general population in England was estimated to be 0.06% in people aged 20-49 years, 0.84% in people aged 50-69 years, and 5.92% in people aged ≥70 years.

*Case fatality rate in the general population: sensitivity analyses*

Based on the lower and upper bounds of the credible intervals published by ONS, the total cases during the 13 weeks from 28 May 2020 to 26 August 2020 could have been between 78,871 and 161,740.

Based on the lower bound estimate for SARS-CoV-2 cases (78,871) it was projected there would have been of 46,840 cases in people aged 20-49 years, 18,226 in people aged 50-69 years, and 13,805 cases in people aged ≥70 years. Therefore, for the best-case scenario (lowest NNV), case fatality rates were calculated as 0.08% in people aged 20-49 years, 1.23% in people aged 50-69 years, and 8.65% in people aged ≥70 years.

Based on the lower bound estimate for SARS-CoV-2 cases (161,740) it was projected there would have been of 96,054 cases in people aged 20-49 years, 37,376 in people aged 50-69 years, and 28,310 cases in people aged ≥70 years. Therefore, for the worst-case scenario (highest NNV), case fatality rates were calculated as 0.04% in people aged 20-49 years, 0.60% in people aged 50-69 years, and 4.22% in people aged ≥70 years.

*Calculation of NNV to prevent one COVID-19-related death over 30 days following vaccination*

To calculate NNV to prevent one death over 30 days following vaccination in the surgical groups:

NNV = 1 / S

S: Postoperative mortality (within 30 days following surgery) attributable to SARS-CoV-2 infection

To calculate NNV to prevent one death over 30 days following vaccination in the general population:

NNV = 1 / (D * 30 * C)

D: Daily community SARS-CoV-2 incidence

C: Case fatality rate for SARS-CoV-2 in the general population

*COVID-19-related deaths prevented*

The number of COVID-19-related deaths that would be prevented in one year preoperatively vaccinating surgical patients rather than age-matched controls was calculated separately for subgroups stratified by age (18-49 years, 50-69 years, ≥70 years) and indication (cancer, non-cancer).

Additional lives saved = ( V * P * M * I * R ) - ( V * P * M * D * 30 * CFR )

V: Total pre-pandemic annual surgical volume

P: Proportion of all surgery that is performed in subgroup of interest

M: Estimated global surgical volume in 2021 as a proportion of pre-pandemic volume

I: Postoperative SARS-CoV-2 rate in the first 30 days following surgery

R Average marginal effect for mortality between patients who did and did not have postoperative SARS-CoV-2 infection

D: Daily community SARS-CoV-2 incidence

C: Case fatality rate for SARS-CoV-2 in the general population

V * P * M produces the absolute global total for subgroup surgical volume over one year.

V * P * M * I * R produces the subgroup annual total for lives saved in the first 30 postoperative days by preoperatively vaccinating surgical patients.

V * P * M * D * 30 * C produces an annual total for lives saved over 30 days by vaccinating the same number of age-matched individuals from the general population.

The pre-pandemic total annual global volume of elective surgery has been estimated as 170,195,382 procedures, including both inpatient and elective procedures. Data from GlobalSurg-CovidSurg Week was used to estimate the proportion of all surgery that is performed in each subgroup of interest (Supplementary Table 5); the denominator for these calculations included both day case patients and patients with preoperative SARS-CoV-2 infection (Supplementary Figure 1).

*Sensitivity analyses for community SARS-CoV-2 incidence*

Daily community SARS-CoV-2 incidence data was available from Our World in Data for 183 of 193 United Nations member states^8^. The 10 countries without SARS-CoV-2 data (Kiribati, Micronesia, Nauru, North Korea, Palau, St. Kitts and Nevis, Timor-Leste, Tonga, Turkmenistan, Tuvalu) were excluded from this analysis.

For each country, average daily community SARS-CoV-2 incidence data was considered from the date when it confirmed 100 cumulative SARS-CoV-2 cases, through to 31 December 2020. Seven countries (Dominica, Fiji, Laos, Marshall Islands, Samoa, Solomon Islands, Vanuatu) recorded fewer than 100 SARS-CoV-2 cases in 2020. These countries were assigned SARS-CoV-2 incidence values equivalent to the country with the lowest average rate (Tanzania: 0.027 cases per million population per day).

Countries were ranked by the calculated average daily SARS-CoV-2 incidence and split into tertiles (Supplementary Table 6). Median daily community SARS-CoV-2 incidence was calculated for each tertile to produce values for low, medium, and high community SARS-CoV-2 incidence. The main analysis was based on medium SARS-CoV-2 incidence (26.48 cases per million population per day). The low (1.54 cases per million population per day) and high (120.56 cases per million population per day) community SARS-CoV-2 incidence rates were used in the sensitivity analyses.

*Best- and worst-case scenarios*

To further explore uncertainty, in addition to the main analysis, best- and worst-case scenarios were produced. The best-case scenario represents the lowest likely value for NNV and the highest likely value for additional COVID-19-related deaths prevented, whereas the worst-case scenario represents the highest likely value for NNV and the lowest likely value for additional COVID-19-related deaths prevented**.**

The best- and worst-case scenarios are based around the key areas of uncertainty:

- Case fatality rate in the general population: NNV will be more favourable (lower NNV) the higher the case fatality rate is. The ONS provides credible intervals for their estimates of SARS-CoV-2 incidence. For the best-case scenario, the lower bounds for the credible intervals were used (thereby reducing the denominator for case fatality rate calculation, producing the highest likely baseline case fatality rate). For the worst-case scenario, the upper bounds for the credible intervals were used.
- Postoperative SARS-CoV-2 rates: 95% confidence intervals were calculated for these rates. For the best-case scenario, upper bounds were used, and for the worst-case scenario, the lower bounds were used.
- Postoperative mortality attributable to SARS-CoV-2: 95% confidence intervals were calculated for the average marginal effects. For the best-case scenario, upper bounds were used, and for the worst-case scenario, the lower bounds were used.
- Vaccine effectiveness: no deaths due to COVID-19 have been reported beyond 1 week following SARS-CoV-2 vaccination in the phase III trials published to date, although these trials were not designed to detect mortality differences^9-12^. In the main analysis vaccines were modelled as having 95% effectiveness. For the best-case scenario effectiveness was modelled as 100%, and for the worst-case scenario effectiveness was modelled as 80%.

The parameters used in the sensitivity analyses are summarised in Table 1.

*Comparison of general population case fatality rate estimates to published literature*

There are few high-quality, age-stratified studies of overall SARS-CoV-2 case fatality rates for the general population, capturing both community and hospital deaths. Regional variation in availability of SARS-CoV-2 testing makes it difficult to calculate reliable case fatality rates using reported SARS-CoV-2 incidence statistics, as these are likely to substantially underestimate the true numbers of cases and therefore overestimate case fatality rates. More accurate incidence data can be obtained from population surveillance studies.

The case fatality rates calculated in this analysis are consistent with previously published estimates from a study based on seroprevalence data from 45 countries. This found case fatality rates to be <0.5% in people aged 20-64 years, around 1% in people aged 65-69 years, around 2-8% in people aged ≥70 years^13^. However, from the publicly available data, it was not possible to obtain overall estimates for case fatality rates in people aged 18-49 years versus 50-69 years versus ≥70 years.

*Comparison of general population NNV estimates to published literature*

Peer-reviewed estimates are not available for NNV for SARS-CoV-2 in the general population. In this analysis there was a pattern of increasing NNV with decreasing age. This is consistent with a previous analysis by the COVID Actuaries Response Group. They produced estimates for NNV based on the number of deaths due to COVID-19 recorded in England from the start of the pandemic to 20 November 2020^14^. They found that the NNV was 20 for vaccination of care home residents, 160 for people aged over 80 years, 350 for people aged over 75 years, 600 for people aged over 70 years, increasing to 47,000 for people aged under 50 years. In the period February to November 2020 the United Kingdom was amongst the top 10 countries in the world for SARS-CoV-2 cases per capita. As NNV varies depending on disease incidence, NNV estimates from England will be lower than global NNV estimates; this explains why NNV values in our analysis were higher.

**References**

1. Office for National Statistics (2020). Coronavirus (COVID-19) Infection Survey pilot: England and Wales, 11 September 2020. Accessed online on 7 January 2021 at <https://www.ons.gov.uk/peoplepopulationandcommunity/healthandsocialcare/conditionsanddiseases/bulletins/coronaviruscovid19infectionsurveypilot/11september2020>.

2. Office for National Statistics. Population estimates. Accessed online (23 January 2021) at <https://www.ons.gov.uk/peoplepopulationandcommunity/populationandmigration/populationestimates>.

3. Intensive Care National Audit & Research Centre (2021). Report on 196 patients critically ill with COVID-19. Accessed online 7 January 2021 at <https://www.icnarc.org/DataServices/Attachments/Download/326bbfc2-d851-eb11-912d-00505601089b>.

4. Jung SM, Akhmetzhanov AR, Hayashi K, et al. Real-Time Estimation of the Risk of Death from Novel Coronavirus (COVID-19) Infection: Inference Using Exported Cases. *J Clin Med* 2020; **9**(2).

5. Verity R, Okell LC, Dorigatti I, et al. Estimates of the severity of coronavirus disease 2019: a model-based analysis. *Lancet Infect Dis* 2020; **20**(6): 669-77.

6. Department of Health and Social Care (2020). Demographic data for coronavirus (COVID-19) testing (England): 28 May to 26 August. Accessed online 7 January 2021 at <https://www.gov.uk/government/publications/demographic-data-for-coronavirus-testing-england-28-may-to-26-august/demographic-data-for-coronavirus-covid-19-testing-england-28-may-to-26-august>.

7. Coronavirus Dashboard. Accessed 11 January 2021 from <https://coronavirus.data.gov.uk>.

8. Ritchie H, Ortiz-Ospina E, Beltekian D, Mathieu E, Hasell J, Macdonald B, Giattino C, Roser M (2021). Coronavirus Pandemic (COVID-19). Accessed online on 11 January 2021 form <https://ourworldindata.org/coronavirus>.

9. Baden LR, El Sahly HM, Essink B, et al. Efficacy and Safety of the mRNA-1273 SARS-CoV-2 Vaccine. *N Engl J Med* 2020.

10. Logunov DY, Dolzhikova IV, Zubkova OV, et al. Safety and immunogenicity of an rAd26 and rAd5 vector-based heterologous prime-boost COVID-19 vaccine in two formulations: two open, non-randomised phase 1/2 studies from Russia. *Lancet* 2020; **396**(10255): 887-97.

11. Polack FP, Thomas SJ, Kitchin N, et al. Safety and Efficacy of the BNT162b2 mRNA Covid-19 Vaccine. *N Engl J Med* 2020; **383**(27): 2603-15.

12. Voysey M, Clemens SAC, Madhi SA, et al. Safety and efficacy of the ChAdOx1 nCoV-19 vaccine (AZD1222) against SARS-CoV-2: an interim analysis of four randomised controlled trials in Brazil, South Africa, and the UK. *Lancet* 2021; **397**(10269): 99-111.

13. O'Driscoll M, Ribeiro Dos Santos G, Wang L, et al. Age-specific mortality and immunity patterns of SARS-CoV-2. *Nature* 2020.

14. Gong Y, McDonald S (2020). How logical is the UK’s vaccine priority ordering? Accessed online 7 January 2021 at <https://www.covid-arg.com/post/vaccine-priorities>.

**Table S1 Overall global 30-day postoperative SARS-CoV-2 rates following inpatient elective surgery**

|  | **Point estimate** | **95% confidence interval** |
| --- | --- | --- |
| Age 18-49 years | | |
| Elective non-cancer surgery | 0.68% (124/18241) | 0.57%-0.81% |
| Elective cancer surgery | 1.00% (36/3595) | 0.70%-1.38% |
| Age 50-69 years | | |
| Elective non-cancer surgery | 0.79% (110/13906) | 0.65%-0.95% |
| Elective cancer surgery | 1.56% (120/7671) | 1.30%-1.87% |
| Age ≥70 years | | |
| Elective non-cancer surgery | 0.87% (69/7932) | 0.68%-1.10% |
| Elective cancer surgery | 1.56% (8/5244) | 1.25%-1.94% |

**Table S2 Unadjusted and adjusted model for 30-day mortality in elective inpatient surgery patients aged 18-49 years**

| **Factor** | **Unadjusted** | | **Adjusted** | |
| --- | --- | --- | --- | --- |
|  | **OR (95% CI)** | **p-value** | **OR (95% CI)** | **p-value** |
| Age | | | | |
| 18-29 years | Reference | - | Reference | - |
| 30-39 years | 0.79 (0.42-1.46) | 0.450 | 0.69 (0.37-1.29) | 0.239 |
| 40-49 years | 1.62 (0.94-2.76) | 0.080 | 0.99 (0.56-1.73) | 0.968 |
| Sex | | | | |
| Female | Reference | - | Reference | - |
| Male | 1.55 (1.00-2.38) | 0.047 | 1.45 (0.93-2.26) | 0.102 |
| ASA | | | | |
| Grades 1-2 | Reference | - | Reference | - |
| Grades 3-5 | 8.36 (5.30-13.17) | <0.001 | 6.69 (4.19-10.69) | <0.001 |
| Indication | | | | |
| Non-cancer surgery | Reference | - | Reference | - |
| Cancer surgery | 4.67 (2.98-7.33) | <0.001 | 3.92 (2.45-6.27) | <0.001 |
| Grade of surgery | | | | |
| Minor | Reference | - | Reference | - |
| Major | 1.55 (0.89-2.68) | 0.121 | 1.19 (0.67-2.10) | 0.546 |
| Postoperative SARS-CoV-2 | | | | |
| No | Reference | - | Reference | - |
| Yes | 5.31 (1.60-17.60) | 0.006 | 4.07 (1.18-14.13) | 0.027 |

ASA: American Society of Anesthesiologists physical status grade; CI: confidence interval; OR: odds ratio

Multilevel models including country and hospital effects were used for both unadjusted and adjusted analyses. The adjusted analysis was adjusted for age, sex, ASA grade, surgical indication, grade of surgery, and postoperative SARS-CoV-2 infection.

**Table S3 Unadjusted and adjusted model for 30-day mortality in elective inpatient surgery patients aged 50-69 years**

| **Factor** | **Unadjusted** | | **Adjusted** | |
| --- | --- | --- | --- | --- |
|  | **OR (95% CI)** | **p-value** | **OR (95% CI)** | **p-value** |
| 50-59 years | Reference | - | Reference | - |
| 60-69 years | 1.44 (1.08-1.91) | 0.013 | 1.22 (0.91-1.64) | 0.190 |
| Sex | | | | |
| Female | Reference | - | Reference | - |
| Male | 1.32 (1.00-1.75) | <0.001 | 1.17 (0.87-1.57) | 0.299 |
| ASA | | | | |
| Grades 1-2 | Reference | - | Reference | - |
| Grades 3-5 | 5.71 (4.16-7.83) | 0.053 | 5.24 (3.79-7.24) | <0.001 |
| Indication | | | | |
| Non-cancer surgery | Reference | - | Reference | - |
| Cancer surgery | 2.32 (1.72-3.12) | <0.001 | 2.07 (1.53-2.82) | <0.001 |
| Grade of surgery | | | | |
| Minor | Reference | - | Reference | - |
| Major | 1.38 (0.96-1.98) | 0.082 | 1.04 (0.72-1.52) | 0.824 |
| Postoperative SARS-CoV-2 | | | | |
| No | Reference | - | Reference | - |
| Yes | 13.67 (7.76-24.08) | <0.001 | 11.52 (6.30-21.09) | <0.001 |

ASA: American Society of Anesthesiologists physical status grade; CI: confidence interval; OR: odds ratio

Multilevel models including country and hospital effects were used for both unadjusted and adjusted analyses. The adjusted analysis was adjusted for age, sex, ASA grade, surgical indication, grade of surgery, and postoperative SARS-CoV-2 infection.

**Table S4 Unadjusted and adjusted model for 30-day mortality in elective inpatient surgery patients aged ≥70 years**

| **Factor** | **Unadjusted** | | **Adjusted** | |
| --- | --- | --- | --- | --- |
|  | **OR (95% CI)** | **p-value** | **OR (95% CI)** | **p-value** |
| Age | | | | |
| 70-79 years | Reference | - | Reference | - |
| ≥80 years | 1.86 (1.41-2.44) | <0.001 | 1.66 (1.25-2.20) | <0.001 |
| Sex | | | | |
| Female | Reference | - | Reference | - |
| Male | 1.56 (1.18-2.06) | 0.002 | 1.51 (1.14-2.01) | 0.004 |
| ASA | | | | |
| Grades 1-2 | Reference | - | Reference | - |
| Grades 3-5 | 5.17 (3.61-7.41) | <0.001 | 4.44 (3.09-6.38) | <0.001 |
| Indication | | | | |
| Non-cancer surgery | Reference | - | Reference | - |
| Cancer surgery | 1.97 (1.49-2.61) | <0.001 | 1.85 (1.39-2.46) | <0.001 |
| Grade of surgery | | | | |
| Minor | Reference | - | Reference | - |
| Major | 1.63 (1.14-2.34) | 0.008 | 1.52 (1.05-2.19) | 0.027 |
| Postoperative SARS-CoV-2 | | | | |
| No | Reference | - | Reference | - |
| Yes | 13.41 (8.20-21.94) | <0.001 | 10.31 (6.18-17.20) | <0.001 |

ASA: American Society of Anesthesiologists physical status grade; CI: confidence interval; OR: odds ratio

Multilevel models including country and hospital effects were used for both unadjusted and adjusted analyses. The adjusted analysis was adjusted for age, sex, ASA grade, surgical indication, grade of surgery, and postoperative SARS-CoV-2 infection.

**Table S5 Estimates for surgical case-mix and pre-pandemic annual volume for elective inpatient surgery**

|  | **Case-mix (95% CI)*** | **Pre-pandemic annual volume** |
| --- | --- | --- |
| Age 18-49 years | | |
| Elective non-cancer surgery | 20.44% (20.18%-20.71%) | 34,794,436 |
| Elective cancer surgery | 4.03% (3.90%-4.16%) | 6,857,410 |
| Age 50-69 years | | |
| Elective non-cancer surgery | 15.59% (15.35%-15.82%) | 26,525,494 |
| Elective cancer surgery | 8.60% (8.41%-8.78%) | 14,632,327 |
| Age ≥70 years | | |
| Elective non-cancer surgery | 8.89% (8.70%-9.08%) | 15,130,181 |
| Elective cancer surgery | 5.88% (5.72%-6.03%) | 10,002,859 |

CI: confidence interval

Case-mix indicates the proportion of all elective surgery that is accounted for by each subgroup. The denominator (170,195,382 surgeries per year) is the pre-pandemic annual global total for all elective surgeries. This includes both daycase and inpatient surgery, and all patients regardless of SARS-CoV-2 status. Annual pre-pandemic volume was calculated for each group using the point estimate for case-mix.

Please note that due to the case-mix estimates presented being rounded, replication of the calculation may lead to different surgical volume values to those reported in the table.

**Table S6 Country-level average daily SARS-CoV-2 incidence rates for 2020**

| **Tertile 1** | | **Tertile 2** | | **Tertile 3** | |
| --- | --- | --- | --- | --- | --- |
| Tanzania | 0.03 | Japan | 5.92 | Albania | 71.24 |
| Dominica* | 0.03 | Ghana | 6.26 | Estonia | 71.81 |
| Fiji* | 0.03 | Antigua and Barbuda | 6.49 | United Arab Emirates | 72.67 |
| Laos* | 0.03 | Kenya | 6.54 | Russia | 73.89 |
| Marshall Islands* | 0.03 | Sri Lanka | 7.13 | Belarus | 74.19 |
| Samoa* | 0.03 | Pakistan | 7.47 | Latvia | 75.4 |
| Solomon Islands* | 0.03 | Algeria | 7.94 | Azerbaijan | 76.73 |
| Vanuatu* | 0.03 | Lesotho | 7.96 | Cabo Verde | 84.53 |
| Vietnam | 0.05 | Uzbekistan | 8.24 | Ukraine | 88.13 |
| Cambodia | 0.06 | Myanmar | 8.91 | Cyprus | 88.13 |
| China | 0.19 | Gambia | 9.29 | Bahamas | 88.65 |
| Yemen | 0.29 | Indonesia | 9.3 | Oman | 89.74 |
| Burundi | 0.31 | Seychelles | 9.41 | Lebanon | 91.02 |
| Thailand | 0.35 | Bangladesh | 11.55 | Denmark | 95.35 |
| Niger | 0.49 | Malaysia | 11.71 | Maldives | 99.97 |
| Papua New Guinea | 0.51 | St. Vincent and the Grenadines | 12.96 | Malta | 101.15 |
| Chad | 0.51 | Mauritania | 13.53 | Jordan | 101.24 |
| Brunei Darussalam | 0.53 | Venezuela | 14.2 | Bulgaria | 101.38 |
| Democratic Republic of the Congo | 0.71 | Equatorial Guinea | 14.69 | Monaco | 101.93 |
| Benin | 1.09 | Philippines | 14.76 | Peru | 106.16 |
| Burkina Faso | 1.12 | Gabon | 16.46 | Chile | 109.08 |
| Somalia | 1.13 | Jamaica | 16.5 | Italy | 111.34 |
| Mauritius | 1.22 | Trinidad and Tobago | 18.53 | Colombia | 111.71 |
| Sierra Leone | 1.27 | Sao Tome and Principe | 18.76 | Romania | 112.15 |
| Liberia | 1.3 | Uruguay | 19.08 | Slovakia | 113.74 |
| South Sudan | 1.31 | Djibouti | 21.68 | Costa Rica | 116.16 |
| Mali | 1.31 | Finland | 22.13 | Hungary | 116.7 |
| Mongolia | 1.49 | St. Lucia | 24.04 | Poland | 116.76 |
| Nigeria | 1.53 | Grenada | 24.44 | Bosnia and Herzegovina | 118.6 |
| New Zealand | 1.54 | India | 25.39 | Kuwait | 120.28 |
| Malawi | 1.54 | El Salvador | 26.48 | United Kingdom | 120.56 |
| Eritrea | 1.73 | Guatemala | 28.94 | Brazil | 122.83 |
| Togo | 1.73 | Norway | 30.32 | Argentina | 125.31 |
| Sudan | 2.27 | Eswatini | 32.47 | Moldova | 126.33 |
| Rwanda | 2.35 | Singapore | 32.57 | France | 133.62 |
| Madagascar | 2.4 | Guyana | 33.6 | Austria | 133.96 |
| Mozambique | 2.53 | Botswana | 33.64 | Spain | 135.21 |
| Angola | 2.59 | Saudi Arabia | 35.55 | Portugal | 137.97 |
| Syria | 2.93 | Nepal | 37.41 | North Macedonia | 140.2 |
| Cote d'Ivoire | 3.03 | Kazakhstan | 38.12 | Sweden | 149.58 |
| Uganda | 3.2 | Mexico | 38.27 | Netherlands | 156.72 |
| Cameroon | 3.55 | Tunisia | 41.57 | Israel | 166.3 |
| Haiti | 3.62 | Ecuador | 41.67 | Qatar | 168.63 |
| Cuba | 3.73 | Morocco | 41.74 | Serbia | 172.39 |
| Australia | 3.74 | Honduras | 44.21 | Switzerland | 173.01 |
| South Korea | 3.81 | Kyrgyz Republic | 44.95 | Croatia | 178.26 |
| Guinea | 3.82 | Greece | 45.28 | Lithuania | 181.09 |
| Comoros | 3.92 | Iran | 47.05 | Belize | 181.65 |
| Nicaragua | 4 | Namibia | 49.43 | Bahrain | 183.19 |
| Senegal | 4.05 | Bolivia | 49.67 | Belgium | 185.31 |
| Ethiopia | 4.19 | Suriname | 49.92 | Armenia | 186.69 |
| Central African Republic | 4.24 | Iraq | 50.33 | Panama | 198.53 |
| Zimbabwe | 4.24 | Canada | 52.3 | Slovenia | 199.71 |
| Zambia | 4.56 | Dominican Republic | 55.02 | United States | 200.02 |
| Egypt | 4.6 | Paraguay | 55.79 | Georgia | 205.73 |
| Afghanistan | 4.74 | Iceland | 56.31 | Czech Republic | 228.23 |
| Bhutan | 4.77 | Turkey | 57.23 | San Marino | 236.93 |
| Republic of the Congo | 4.89 | South Africa | 61.67 | Luxembourg | 255.26 |
| Guinea-Bissau | 4.89 | Ireland | 63.37 | Montenegro | 277.8 |
| Barbados | 5.7 | Libya | 66.88 | Andorra | 361.53 |
| Tajikistan | 5.7 | Germany | 68.67 | Liechtenstein | 410.87 |
| Tertile 1 median | 1.54 | Tertile 2 median | 26.48 | Tertile 3 median | 120.56 |

Figures reported as averaged daily SARS-CoV-2 cases per million population per day from the date when the country confirmed 100 cumulative SARS-CoV-2 cases, through to 31 December 2020.

*Seven countries recorded fewer than 100 SARS-CoV-2 cases in 2020. These countries were assigned SARS-CoV-2 incidence values equivalent to the country with the lowest average rate.

**Table S7 Additional COVID-19-related deaths prevented within 30 days of surgery if surgical patients were vaccinated preoperatively in preference to age-matched controls from the general population**

| **2021 surgical volume as proportion of pre-pandemic annual volume** | **18-49 years** | **50-69 years** | **≥70 years** |
| --- | --- | --- | --- |
| 25% | 856  (0-2,967) | 9,831  (3,437-18,566) | 8,875  (3,288-16,803) |
| 50% | 1,713  (0-5,934) | 19,663  (6,875-37,131) | 17,749  (6,576-33,607) |
| 75% | 2,569  (0-8,900) | 29,494  (10,312-55,697) | 26,624  (9,865-50,410) |
| 100% | 3,425  (0-11,867) | 39,325  (13,750-74,262) | 35,498  (13,154-67,213) |

Figures presented based on a range of different projections for 2021 surgical volume as a proportion of pre-pandemic volume. Estimates are based on the medium community SARS-CoV-2 infection rates. Ranges in parentheses indicate results from worst- and best-case scenarios.

**Fig. S1 Flowchart for inclusion of GlobalSurg-CovidSurg Week data**

ASA: American Society of Anesthesiologists physical status grade

*This denominator was used for estimating case-mix

Analysis included patients whose data was available as of 4 February 2021.
